# Supplementary material for: Rotational fishing enables biodiversity recovery and provides a model for oyster (Ostrea edulis) habitat restoration
Source: PLoS One. 2023 Mar 29;18(3):e0283345. doi: 10.1371/journal.pone.0283345 (PMC10058151; doi:10.1371/journal.pone.0283345)
Supplement: S1 Table — Average oyster shell density and percentage cover data for treatments 1,2 & 3. (DOCX) [file pone.0283345.s002.docx]

Table 1. Species abundance data from Treatment 1, 2 & 3.

| **Species** | Species Abundance | | | | | | | | | | | |
| --- | --- | --- | --- | --- | --- | --- | --- | --- | --- | --- | --- | --- |
|  | Treatment 1 | | | | Treatment 2 | | | | Treatment 3 | | | |
|  | **1.1** | **1.2** | **1.3** | **1.4** | **2.1** | **2.2** | **2.3** | **2.4** | **3.1** | **3.2** | **3.3** | **3.4** |
| Pagurus bernhardus | 0 | 3 | 0 | 28 | 15 | 15 | 12 | 19 | 23 | 15 | 20 | 28 |
| Pagurus cuanensis | 0 | 0 | 0 | 0 | 0 | 1 | 0 | 0 | 0 | 0 | 0 | 0 |
| Hyas araneus | 0 | 0 | 0 | 1 | 2 | 0 | 0 | 2 | 4 | 6 | 3 | 4 |
| Carcinus maenas | 1 | 0 | 0 | 1 | 0 | 0 | 1 | 0 | 0 | 5 | 3 | 0 |
| Liocarcinus depurator | 0 | 1 | 1 | 2 | 1 | 1 | 0 | 10 | 2 | 2 | 3 | 2 |
| Liocarcinus holsatus | 0 | 0 | 0 | 0 | 0 | 0 | 0 | 0 | 0 | 1 | 1 | 0 |
| Necora puber | 0 | 0 | 0 | 0 | 0 | 0 | 0 | 0 | 0 | 1 | 0 | 0 |
| Cancer pagurus | 0 | 0 | 0 | 0 | 0 | 0 | 0 | 1 | 0 | 2 | 0 | 0 |
| Macropodia rostrata | 0 | 0 | 0 | 0 | 3 | 0 | 1 | 0 | 0 | 0 | 0 | 0 |
| Inachus dorsettensis | 0 | 0 | 0 | 2 | 0 | 2 | 0 | 3 | 2 | 4 | 0 | 2 |
| (Juv) Munida sp. | 0 | 0 | 0 | 0 | 0 | 0 | 0 | 0 | 0 | 1 | 0 | 1 |
| Crangon crangon | 0 | 0 | 0 | 0 | 1 | 1 | 2 | 0 | 0 | 0 | 1 | 1 |
| Palaemon serratus | 0 | 0 | 0 | 0 | 0 | 0 | 0 | 0 | 0 | 0 | 1 | 0 |
| Pantopoda sp. | 0 | 0 | 0 | 0 | 0 | 0 | 0 | 0 | 0 | 1 | 0 | 1 |
| Antedon bifida | 0 | 0 | 0 | 11 | 8 | 5 | 5 | 50 | 42 | 1 | 2 | 6 |
| Crossaster papposus | 0 | 0 | 0 | 0 | 0 | 0 | 1 | 0 | 1 | 0 | 3 | 1 |
| Marthasterias glacialis | 0 | 0 | 0 | 0 | 0 | 0 | 0 | 0 | 0 | 2 | 0 | 0 |
| Henrica oculata | 0 | 0 | 0 | 0 | 1 | 0 | 0 | 1 | 1 | 0 | 2 | 0 |
| Ophiothrix fragilis | 0 | 0 | 0 | 0 | 0 | 1 | 0 | 1 | 3 | 9 | 0 | 3 |
| Syngnathus acus | 0 | 0 | 0 | 1 | 0 | 0 | 0 | 0 | 0 | 0 | 0 | 1 |
| Limanda limanda | 1 | 0 | 0 | 0 | 0 | 0 | 0 | 0 | 0 | 0 | 0 | 0 |
| Callionymus reticulatus | 1 | 0 | 0 | 0 | 0 | 0 | 0 | 0 | 0 | 0 | 0 | 0 |
| Callionymus lyra | 0 | 0 | 0 | 1 | 0 | 0 | 1 | 0 | 0 | 0 | 0 | 0 |
| Gobius niger | 1 | 0 | 1 | 3 | 0 | 4 | 1 | 11 | 5 | 0 | 2 | 4 |
| Pomatoschistus microps | 8 | 1 | 2 | 6 | 11 | 8 | 1 | 7 | 2 | 14 | 3 | 3 |
| Pholis gunnellus | 0 | 0 | 0 | 0 | 0 | 0 | 0 | 1 | 0 | 0 | 0 | 0 |
| Tauras bubalis | 0 | 0 | 0 | 0 | 0 | 0 | 0 | 1 | 0 | 0 | 0 | 0 |

Table 2. Average oyster shell density and percentage cover data for treatments 1,2 & 3.

| Treatment | Sample | Oyster shell density (oysters/0.25m²) | Oyster shell density (oysters/m²) | Oyster shell percentage cover (%) |
| --- | --- | --- | --- | --- |
| 1 | 1.1 | 2.1 | 8.4 | 1.13 |
|  | 1.2 | 2.2 | 8.7 | 0.89 |
|  | 1.3 | 0.4 | 1.5 | 0.16 |
|  | 1.4 | 3.8 | 15.3 | 1.15 |
| 2 | 2.1 | 7.7 | 30.9 | 2.95 |
|  | 2.2 | 4.4 | 17.6 | 1.45 |
|  | 2.3 | 3.2 | 12.9 | 1.52 |
|  | 2.4 | 6.0 | 24.0 | 2.10 |
| 3 | 3.1 | 13.5 | 53.8 | 7.59 |
|  | 3.2 | 31.7 | 126.8 | 20.01 |
|  | 3.3 | 19.0 | 76.0 | 11.93 |
|  | 3.4 | 7.7 | 30.7 | 4.70 |
