# Supplementary material for: Rotational fishing enables biodiversity recovery and provides a model for oyster (Ostrea edulis) habitat restoration
Source: PLoS One. 2023 Mar 29;18(3):e0283345. doi: 10.1371/journal.pone.0283345 (PMC10058151; doi:10.1371/journal.pone.0283345)
Supplement: Attachment — Submitted filename: Response to reviewers.docx [file pone.0283345.s003.docx]

Dear José A. Fernández Robledo, Ph.D. 05/09/22

Thank you for giving me the opportunity to submit a revised draft of my manuscript titled

*Rotational fishing enables biodiversity recovery and provides a model for oyster (Ostrea edulis) habitat restoration* to Plos One. We appreciate the time and effort that you and the reviewers have dedicated to providing your valuable feedback on my manuscript. We are grateful to the reviewers for their insightful comments on my paper. We have been able to incorporate changes to reflect most of the suggestions provided by the reviewers. We have highlighted the changes within the manuscript.

See the following table for a point-by-point response to the reviewers’ comments and concerns.

| **Line** | **Comment** | **Response** | **New line** |
| --- | --- | --- | --- |
| Acknowledgements | Funders should not be listed in the acknowledgement | Removed funders from acknowledgements | n/a |
| Title page | Please ensure that you include a title page within your main document. You should list all authors and all affiliations as per our author instructions and clearly indicate the corresponding author. | Corrected | n/a |
| Figure 1 | Copyright | Map was created using ArcGIS using shapefiles from ESRI.  Esri UK, Esri, 2022, Garmin, GeoTechnologies, Inc., USGS \| Esri, 2022]) | n/a |
| Figure 2 | Consent for photo | Photo is of Scientific Divers conducting survey. Not of study subjects. | n/a |
| Figure 3 | Refer to figure in text | Corrected | 219 |
| Supporting files | Include captions for supporting information files at the end of the manuscript http://journals.plos.org/plosone/s/supporting-information | Corrected | n/a |
| 22 | Change to “percent cover”--also, is this percent cover of benthos that is shells? To make clearer, the authors could consider changing this metric to ‘benthic shell cover’? | Changed to “Oyster shell density, oyster shell percentage cover” as specific to oyster shell cover | 27 |
| 23 | Since there are multiple plots within each treatment, I would replace the word “plot” with treatment. If the authors made this edit they could then rephrase the following sentence to “Shell density was 8.5 times higher in the six-year treatment compared to the one-year treatment, whilst…”. | Corrected |  |
| 31 | Rephrase slightly, perhaps by starting with the result and deleting the first section of the sentence and start with something like-- “Here we show that the likely scale of biodiversity gain….”. The authors end with how this can be used for conservation and management so the first half of the sentence is unnecessary | Changed to “The findings from the present study indicate the likely scale of biodiversity gain in oyster habitat restoration and a cost-effective tool (shell density) to judge progress in restoration projects.” | 36 |
| 35 | I suggest the authors open with a broader introduction to restoration and the need for reference sites, data, etc. This would provide more context for the first paragraph which would follow and would make the paper applicable to a broader audience. This is a huge issue/topic of interest in the field of restoration and could be fleshed out more. The authors could then use their study to help provide that reference data that is so critical for evaluating project outcomes. | Changed to:  “Habitat and biodiversity restoration is becoming an increasingly popular conservation practice around the world in both terrestrial and aquatic habitats [1][2][4][5]. Many of these habitats have been lost or degraded due to anthropogenic activities [3], making them rare or endangered. For restoration to work effectively there must be an ecological understanding of the original habitat, but in many cases, due to the degradation or rarity of the habitat, such studies are not available. A reference habitat (analogous in terms of community composition, niche fulfilment and provision of ecosystem services)[6] can provide a baseline against which restorative progress can be judged and expectations of biodiversity outcomes clarified (see [7]).” | 40 |
| 35 | Define reference model. “Reference” is typically reserved for a natural, un-impacted site but no reference was mentioned in the abstract so from this point on the reader could be thinking that the 6-year treatment is the reference. If there is a reference site included, that should be mentioned in the abstract as the “target”. Create | “A restoration practice that has become common in coastal conservation projects in Europe is that of the native oyster (Ostrea edulis) [1][8]. Due to the rarity of intact O. edulis reef habitats (e.g., [8][9][10][11]) there is a lack of knowledge of their composition and biodiversity [2]. It has therefore been necessary to refer to analogous habitats to fill knowledge gaps and build an understanding of a restored oyster reef.” | 48 |
| 40 | What does “familiar” mean here? I would rephrase. Who is the audience for this paper? If managers, as was set up in the abstract, these individuals would know of rocky reefs biogenic reefs. Also, since most bivalves require some hard substrate upon which to settle, maybe rephrase to emphasize instead that reef structural complexity can be constructed from bedrock, but (often overlooked) bivalves too can provide (more?) intricate microhabitats. | Deleted this line. |  |
| 46-49 | Strongly encourage the authors to integrate natural, undisturbed reefs into the abstract given that this has been the focus thus far of the introduction. | Corrected |  |
| 56-58 | Rephrase to make more concise. Unless introducing monetary value of the services, I’d rephrase to “importance”. Something like “The importance of ecosystem services supported by bivalve-built reefs is gaining recognition by government entities and industry.” It would be great if the authors could point to an example of this increased recognition, there are certainly numerous examples. Perhaps the authors could cite a few papers that lay out examples that support the sentence. When editing this sentence, I recommend the authors remove the last portion of the sentence “for the benefits…” since that is implied when mentioning ecosystem services in the beginning of the sentence. | “In addition to providing physical niches for other species, biogenic shellfish reefs perform other provisioning, supporting and regulating roles [2][17][18][19]. These include bio-deposition [20][21] and providing essential fishery habitat [14][15]. The importance of ecosystem services supported by bivalve reefs are gaining recognition by government entities and industry [2][22]. This is also reflected by the increased investment by companies and NGOs into restoration projects. For example the Glenmorangie Whisky Company, in partnership with the Marine Conservation Society and Heriot-Watt University have established the Dornoch Environmental Enhancement Project [13][23]. This initiative aims to restore native oysters to the Dornoch Firth whilst enhancing biodiversity and water quality in the system: the oyster filter feeding is expected to account for organic waste from the distillery’s discharge water.” | 61 |
| 59-61 | Would benefit from references about the risks to shellfish reefs. Also surprised that climate change (and synergistic effects) not listed among the risks in the long-term | Corrected | 72 |
| 79 | Rephrase to something like “Coincidence with the increased understanding of how important and vulnerable biogenic shellfish reefs are, actions to protect this now rare habitat are now also on the rise.” | “Due to the increased understanding of how importance and vulnerable biogenic shellfish reefs are, actions to protect these now rare habitats are on the rise.” | 90 |
| 81 | I would clarify that oyster restoration is a relatively new approach--in terrestrial and (less so) marine habitats, it is not a new approach. If the authors choose to keep this sentence as is, I suggest clarifying the conservation/management strategy they are comparing restoration to that validates restoration as “relatively new”. I encourage the authors to re-write this sentence since I do not think it is accurate, as written. | Worked in to beginning of the introduction. | 35 |
| 83 | Sometimes restoration plans include plans for chronic intervention (if necessary) so I would add “often” to the sentence--“This often requires building a self-sufficient…”. Additionally, a sentence on passive restoration to encourage natural recovery could be included here. | Corrected |  |
| 85 | Topic sentence is very similar to previous sentences above. I would rephrase. | “The significance of restoration projects are now acknowledged at an international level with support from industry, non-governmental organisations and by governments” | 97 |
| 93 | Remove first clause of sentence, start paragraph with “Oyster restoration is…” | Corrected |  |
| 98 | Replace “articulate” with “re-construct” and revise sentence--“…it is extremely difficult to re-construct the biodiversity characteristics of a fully restored reef, given how rare pristine habitats have become” | Corrected |  |
| 99-101 | Rephrase to better transition from previous sentence. It’s my understanding that the authors are trying to state that studies that compare biodiversity in restored and less disturbed/somewhat natural habitats is necessary for measuring progress towards reaching biodiversity goals. I would re-write this last sentence to reflect this study goal, that will also lead into study aims nicely. | “By examining the biodiversity of recovering oyster habitats this ecological understanding can be gained [2][41] and used to manage expectations of biodiversity outcomes and to measure progress towards biodiversity goals.” | 109 |
| 106 | Rewrite slightly--Something like…“The implications of this work extend from identifying likely expectations for biodiversity development post-restoration to the management of fished biogenic habitats and the biodiversity they support.” | “The implications of this work extend from identifying likely expectations for biodiversity development during and after restoration activities to the management of fished biogenic habitats and the biodiversity they support.” | 116 |
| 111 | Could rephrase Hypothesis 2 to clarify language -- “Recovery of macrofaunal community biodiversity is influenced by time since disturbance”  Hypothesis Question: The authors have not defined biodiversity yet and this term is often overused or misused. It may be nice to define this in the introduction so the reader is aware ahead of time whether you are talking about biodiversity as species richness and evenness (using H’) or some other variable like species density, which is being used as a proxy for biodiversity.  Since biodiversity is a key part of this study, the authors could add more to the introduction and discussion as to why biodiversity is ecologically important. Provide specific examples, cite relevant literature, etc. The majority of the intro was focused on ecosystem services and the importance of biogenic habitats but the ecological importance of the diverse assemblage of species that these habitats support could be strengthened and made more prominent in the intro. | “Biodiversity recovery of the macrofaunal community associated with an oyster reef is influenced by the length of time since disturbance.” | 121 |
| 115-117 | The manuscript would benefit from a clearer description of the methods used in a few areas. Specifically:  Lines 115-117 (study site) - Suggest adding more site description. For instance - What are the depths of the reefs being surveyed? Other than a rotational system, how does the Loch Ryan Oyster Fishery Company operate (e.g., gear type, season, etc.)? | “The fishery has been run by the Wallace family since 1701 and the fishery vessel uses a single dredge which only harvests the animals in the winter months, to avoid the spawning season. The oyster bed is divided into six plots which are harvested in a six-year rotation [42] and were regarded as treatments in the present study.” | 127 |
| 118 | I’d remove the term “plots” and only refer to them as Treatment 1, 2, and 3, each with four replicate transects. Ideally, the authors would have tracked each Treatment as it evolved since there may be differences between treatments unrelated to treatment (i.e., distance to shore, hydrology, relief of bedroom, depth, turbidity, etc.).  Authors should address the above (and other) potential confounding factors. If these factors were not evaluated during the study period, their omission should be acknowledged as a potential explanation that could weaken the strength of the findings. Looking at Figure 1 the Treatments are not interspersed and are clumped, so the potential confoundment of the results due purely to environmental factors and not treatment needs to be addressed (if the data was not collected and shown to be similar across treatments). If this environmental data exists, it should be highlighted in the section that outlines the details for all the various treatments.  Is there no reference habitat? Is Treatment 3 serving as the reference habitat? Since this was a prominent topic in the introduction, I suggest aligning the experimental design to the motivation outlined in the intro. | “Sampling was conducted along a geographic gradient parallel to the shore where environmental conditions were not expected to significantly vary.”  “In keeping with most biodiversity studies, the sampling method presented here is expected to represent the patterns in the entirety of the community. Potential confounding environmental factors, were not included in the study. However as fishing is the dominant structural influence on the community the results from the study are out of proportion with any environmental gradients that would produce this scale of change. Furthermore, the scale and trend in the observed relationships are consistent with successional differences not spatial patterns.” | 134  217 |
| 120-121 | The four replicate transects in each plot are arranged quite differently (treatment 1 – in a rectangle, treatment 2 – in a line spread out, treatment 3 – clustered together). How were the locations of the four transects in each plot selected, and what if any impact might that have on the results? Do the characteristics of each transect vary at all? | Co-ordinate mistake was made -> see updated figure 1 |  |
| 124 | Why were quadrats placed randomly instead of at some standardized interval? Could the authors elaborate on this? Were they opportunistically sampling along the transect line on whatever side had the most biogenic habitat? I suggest the authors justify the sampling design. | “to avoid spatial auto-correlation” | 139 |
| 123-125 | Clearer description of the photo quadrat layout. The picture of the quadrats in figure 2 depicts a grid of 25 quadrats. Were a random sample of those selected at each location along the transect, or just one .5x.5m quadrat photographed/counted at each location, randomly selected along the 25m transect? | “These data were then averaged for each transect in each treatment.” | 143 |
| Figure 3 | is missing axis labels/titles, making it hard to interpret | MDS plot is a 2D spatial representation therefore axis are not normally included. If it is felt necessary the axis would only be labelled as “axis 1” & “axis 2”. |  |
| 142-143 | I’ll defer to the other reviewers here to verify, but I don’t believe Pielou’s evenness should be run on transformed data. If the data were transformed for evaluating evenness, what was the justification? | “…species abundance data were first square-root transformed before being used to calculate Shannon-Wiener’s diversity (H’) and Margalef’s richness (d) biodiversity indices.” | 160 |
| 189-190 | What is the relationship between shell cover and diversity/richness? Reported as strongly related but the correlation/significance is not specified. | “were found to be positively correlated to both shell density and shell cover” | 206 |
| Line 198 | Where did the recovery trajectory of ten years come from? Is this from the literature (if so, needs citation) or the study? | “with a recovery trajectory based on logarithmic models (Fig 5.) of approximately ten years” | 215 |
| 198 | How are the authors defining recovery trajectory? The curve plateauing? What if the plateau in the fished areas is still below what would have been the biodiversity “target” of a natural undisturbed oyster reef? This has not been addressed yet but is critical. If ten years is the “recovered” state, Fig 4 should extend to 10 like Fig 5 does since shell density, which covaries with time since harvesting, is stated as the ultimate cause of increased biodiversity. | “Reef recovery likely begins as early as one year after fishing disturbance and increases over time as oyster spat settles and the consequential density and complexity of oyster shell increases. The recovery time (with sufficient larval supply) for O. edulis biogenic reefs (Figure 7; Figure 8) is potentially decadal in nature [26][47] because O. edulis as the biogenic ecosystem engineer, takes around three to five years to reach maturity [3]. Beyond the six year timeline examined here the biodiversity trajectory is expected to plateau out, but there is would be a need to examine oysters in an undisturbed sate to confirm this. Elsewhere in longer term marine reserves studies, ‘trophic cascades’ have occurred when re-established higher trophic level species have restructured benthic communities beyond simple biodiversity and biomass increases that were initially observed [53]. Although a six year timeline is too short to exclude either this or an ‘intermediate disturbance’ biodiversity pattern [54], as the reef is undergoing physical structural recovery these are unlikely.” | 239 |
| 200 | The authors have yet to acknowledge that what they may be witnessing is a version of the intermediate disturbance hypothesis. What if natural reefs have lower biodiversity? It’s important to acknowledge that the 10-year “recovery” is based on a disturbed system. The authors should include text that acknowledges 1) no reference included, 2) confounding factors could explain differences in biodiversity, 3) have not tracked individual treatments over time, only a snapshot in time so can’t rule out #2. | See above response. | 239 |
| 206 | I don’t think this is an issue at all actually since the level of detail in sampling was the same across all treatments. | “In keeping with most biodiversity studies, the sampling method presented here is expected to represent the patterns in the entirety of the community.” | 219 |
| 213-215 | Minor clarification, would suggest adding "after 6 years" after "allowing macrofauna biodiversity to recover by 60%". As currently written, could be misinterpreted to mean that biodiversity recovers by 60% after each fishing season. | Corrected |  |
| 224 | Why inevitably? | “is potentially decadal in nature” | 242 |
| 225 | What is several years? 2-3 or 5-6? Please specify. | “takes around three to five years” | 243 |
| 223-225 | Is this where the ten years comes from? |  |  |
| 223-225 | The study results show that recovery begins as early as 1 year post-harvest and increases over time as shell density increases, but without knowing what biodiversity would be without any harvest, it’s unclear that the recovery would be on a ten year cycle when the fished system is on a 6-year cycle. Oysters would be recruiting throughout that 6-year period and the contributions of each cohort to overall shell density would be staggered. It’s also possible there is a benefit to biodiversity when the size structure of the oyster reef is varied and that’s what’s driving the pattern (another confounding factor that should be mentioned in the discussion). Treatment 3 is going to have a broader size frequency distribution than Treatment 2 or 1. Since this data was not collected, the contributions of a reef composed of different sized individuals should be addressed.  Figure comment: Have symbols match across all figures, when applicable. | “The recovery of bivalves is dependent on successful larval settlement, recruitment, and growth, suggesting the need to also maintain overall adult stock levels as a source in any restoration or sustainable management scenario. Reef recovery likely begins as early as one year after fishing disturbance and increases over time as oyster spat settles and the consequential density and complexity of oyster shell increases. The recovery time (with sufficient larval supply) for O. edulis biogenic reefs (Figure 7; Figure 8) is potentially decadal in nature [26][47] because O. edulis as the biogenic ecosystem engineer, takes around three to five years to reach maturity [3].” | 237 |
| 223 | remove “aspects of the” | Corrected |  |
| 237-240 | Could this be due to the down-weighting of abundances when transformed? Please clarify whether the data used for the evenness index was transformed. If it was transformed, justify. If it was not transformed, include text that distinguished what data was vs was not transformed. | Corrected |  |
| 261 | Is 1701 the age of the Loch Ryan Company? If so, that detail should be in Intro or Methods. | Corrected |  |
| Data availability | Data on percent oyster shell cover were not available, but the remaining data were. | Updated supporting data |  |

In addition to the above comments, all spelling and grammatical errors pointed out by the

reviewers have been corrected.

We look forward to hearing from you in due time regarding our submission and to respond to

any further questions and comments you may have.

Sincerely,

Naomi Kennon

05/09/22
