# Supplementary material for: Rotational fishing enables biodiversity recovery and provides a model for oyster (Ostrea edulis) habitat restoration
Source: PLoS One. 2023 Mar 29;18(3):e0283345. doi: 10.1371/journal.pone.0283345 (PMC10058151; doi:10.1371/journal.pone.0283345)
Supplement: Attachment — Submitted filename: PONE-D-22-14818.R1_comments_2022-10-01.docx [file pone.0283345.s004.docx]

PONE-D-22-14818.R1 Reviewer Comments

"Rotational fishing enables biodiversity recovery and provides a model for oyster (*Ostrea edulis*) habitat restoration"Top of FormBottom of Form

09-30-2022

The authors addressed all of the comments provided in the first round of peer-review. The clarity and transparency of the revised paper has improved. Provided below are additional comments and suggested edits.

Figure 2 - Consent for photos is typically for those pictured in the photo. I believe PLOS ONE policy is that those scientific divers submit a consent form for their photo to be included in the paper. An exception to this may be if the scientific divers are also co-authors on the manuscript. I would defer to the Editor on this though.

Line 13: Rephrase, shouldn’t end the sentence with “in”. I suggest, “European oyster habitat are now considered rare and there are few examples of undisturbed reefs from which restoration sites could be compared.”

Line 15: I suggest rephrasing--I don’t consider oyster restoration a “feature” or marine conservation practice. Perhaps rephrase to…”…oyster restoration efforts are on the rise, becoming a more prominent component of Europe’s portfolio for marine conservation practices” or something along those lines?

Revised Line 35: I appreciate the authors editing the introduction to make it more applicable to a broader audience. I have indicated line edits in all caps to the revised text below. Deleted “making them rare or endangered”. The authors could emphasize that rarely do we have the data or historical knowledge of the pre-degraded state to set a desired target for a restored habitat. Often, the best we can do is compare restored habitats to ambient conditions of nearby, less impacted, sites (reference sites). I suggest the author re-phrase the third sentence--it’s often a lack of data (not only studies) that make it difficult to determine what condition/historical state you are targeting for restoration. I attempted to provide an option for revision to better connect the last two sentences in the revised paragraph, the authors should review and consider how to best communicate their point here which I believe to be that 1) success requires a fundamental understanding of how a particular ecosystem functions, 2) we lack the data necessary to understand how a restored habitat should function given many of these habitats are rare or degraded and don’t function how they “should”, 3) the best we can do is compare a restored habitat to a nearby reference habitat that for the location, local stressors, etc. serves as the most appropriate target for evaluating restoration outcomes.

“Habitat and biodiversity restoration is becoming an increasingly popular conservation practice around the world in both terrestrial and aquatic habitats [1][2][4][5]. Many of these habitats have been lost or degraded due to anthropogenic activities [3]. EFFECTIVE RESTORATION REQUIRES an ecological understanding of the TARGETED habitat, but in many cases, due to the degradation or rarity of the habitat, WE LACK THE DATA NEEDED TO DETERMINE THE DESIRED CONDITION AND ECOLOGICAL FUNCTIONING FOR A RESTORED HABITAT. A reference habitat (analogous in terms of community composition, niche fulfilment and provision of ecosystem services)[6] can provide a baseline against which restorative progress can be judged and expectations of biodiversity outcomes clarified (see [7]).”

Line 48: Common since when? I would rephrase this sentence. It could be made more clear. I would start with something like “In Europe, native oyster restoration projects have been increasing over the past XYZ”.

By providing the approximate time from which oyster restorations became more common, the authors will get at their earlier point in their original draft that it is a relatively new field that lacks data.

Line 50: Composition of what? Species composition? How is this different from biodiversity in many ways they are components of one another. Perhaps add in a reference that highlights the lack of knowledge on other reef functions (i.e. water filtration, habitat provisioning) and replace “composition” with whatever function you’d like to highlight along with biodiversity.

Line 69-70: Replace “to account for” with “mitigate the impacts of organic waste from the distillery’s discharge water”.

Line 90: Revise. “importance” should be “important”

Line 109: Revise. The revised sentence is still disjointed and hard to follow. What is an ‘ecological understanding’? The authors use this phrase frequently throughout but are mostly concerned with biodiversity. The authors could clarify by better communicating the link between biodiversity and ecosystem functioning. The language is very broad here and should be revised.

Line 139: Authors should state in the methods that the sampling was opportunistic.

Line 143: A supplemental figure of the transect survey layout would be helpful.

Line 217: “Furthermore, the scale and trend in the observed relationships are consistent with successional differences (ADD CITATION HERE) not spatial patterns.”

Figure 3: Add axis 1 and axis 2 labels as suggested by authors.

Line 217: List/acknowledge potential confounding factors that were not co-monitored in this sentence “Potential confounding environmental factors (LIST HERE), were not included in the study.”.

Line 217: Explain what the authors mean by “out of proportion” with any environmental gradients? Also, this added language makes many sweeping statements without acknowledging what environmental gradients, factors, etc. may be at play. The authors should add specificity to the main text about the potential confounding factors that they did not evaluate.

Line 219: Add citations after “In keeping with most biodiversity studies,…”

Line 239: Remove the word “consequential”. Revise “is potentially decadal in nature”--this language could be much improved. The authors should revise this section slightly for conciseness. The data supports that reef recovery begins within a year post disturbance and reaches reference levels within ten years.
