# Supplementary material for: Rotational fishing enables biodiversity recovery and provides a model for oyster (Ostrea edulis) habitat restoration
Source: PLoS One. 2023 Mar 29;18(3):e0283345. doi: 10.1371/journal.pone.0283345 (PMC10058151; doi:10.1371/journal.pone.0283345)
Supplement: Attachment — Submitted filename: Response to reviewers.docx [file pone.0283345.s006.docx]

Dear José A. Fernández Robledo, Ph.D. 10/12/22

Thank you for giving us an opportunity to submit a revised draft of our manuscript *Rotational fishing enables biodiversity recovery and provides a model for oyster (Ostrea edulis) habitat* *restoration* to PLOS One.

We again appreciate the time and effort that you and the reviewers have dedicated to providing your valuable feedback on my manuscript. We are grateful to the reviewers for their insightful comments on the paper and have been able to incorporate changes to reflect the suggestions provided by the reviewers.

See the following table for a point-by-point response to the reviewers’ comments and concerns.

| **Line** | **Comment** | **Response** | **New line** |
| --- | --- | --- | --- |
| Figure 2 | Consent for photos is typically for those pictured in the photo. I believe PLOS ONE policy is that those scientific divers submit a consent form for their photo to be included in the paper. An exception to this may be if the scientific divers are also co-authors on the manuscript. I would defer to the Editor on this though. | Divers are the authors. Relevant authors initials have been included in the figure caption. | Fig 2 |
| 13 | Rephrase, shouldn’t end the sentence with “in”. I suggest, “European oyster habitat are now considered rare and there are few examples of undisturbed reefs from which restoration sites could be compared.” | The reviewers point is accepted. However, insertion of “few examples of” does not represent the situation know to the authors, who don’t believe *any examples* are known*.* We have dealt with the reviewers point by removing “in”. Overall, we feel that this frames the present study appropriately. | 13 |
| 15 | I suggest rephrasing--I don’t consider oyster restoration a “feature” or marine conservation practice. Perhaps rephrase to…”…oyster restoration efforts are on the rise, becoming a more prominent component of Europe’s portfolio for marine conservation practices” or something along those lines? | Revision accepted, rephrased to: “As more is understood of the ecosystem services provided by the reefs, oyster restoration efforts are on the rise, becoming a more prominent component of Europe’s portfolio for marine conservation practices.” | 15 |
| 35 | I appreciate the authors editing the introduction to make it more applicable to a broader audience. I have indicated line edits in all caps to the revised text below. Deleted “making them rare or endangered”. The authors could emphasize that rarely do we have the data or historical knowledge of the pre-degraded state to set a desired target for a restored habitat. Often, the best we can do is compare restored habitats to ambient conditions of nearby, less impacted, sites (reference sites). I suggest the author re-phrase the third sentence--it’s often a lack of data (not only studies) that make it difficult to determine what condition/historical state you are targeting for restoration. I attempted to provide an option for revision to better connect the last two sentences in the revised paragraph, the authors should review and consider how to best communicate their point here which I believe to be that 1) success requires a fundamental understanding of how a particular ecosystem functions, 2) we lack the data necessary to understand how a restored habitat should function given many of these habitats are rare or degraded and don’t function how they “should”, 3) the best we can do is compare a restored habitat to a nearby reference habitat that for the location, local stressors, etc. serves as the most appropriate target for evaluating restoration outcomes. “Habitat and biodiversity restoration is becoming an increasingly popular conservation practice around the world in both terrestrial and aquatic habitats [1][2][4][5]. Many of these habitats have been lost or degraded due to anthropogenic activities [3]. EFFECTIVE RESTORATION REQUIRES an ecological understanding of the TARGETED habitat, but in many cases, due to the degradation or rarity of the habitat, WE LACK THE DATA NEEDED TO DETERMINE THE DESIRED CONDITION AND ECOLOGICAL FUNCTIONING FOR A RESTORED HABITAT. A reference habitat (analogous in terms of community composition, niche fulfilment and provision of ecosystem services)[6] can provide a baseline against which restorative progress can be judged and expectations of biodiversity outcomes clarified (see [7]).” | Revision accepted, rephrased to: “Habitat and biodiversity restoration is becoming an increasingly popular conservation practice around the world in both terrestrial and aquatic habitats [1][2][3][4]. Many of these habitats have been lost or degraded due to anthropogenic activities [5], making them rare or endangered. Effective restoration requires an ecological understanding of the targeted habitat, but in many cases, we lack the data needed to determine the desired condition and biodiversity characteristics for a restored habitat. A reference habitat (analogous in terms of community composition, niche fulfilment and provision of ecosystem services)[6] can provide a baseline against which restorative progress can be judged and expectations of biodiversity outcomes clarified (see [7]).”  (Following revision for line 109, the term ‘ecological functioning’ has been replaced with ‘biodiversity characteristics’ to improve clarity.) | 44 |
| 48 | Common since when? I would rephrase this sentence. It could be made more clear. I would start with something like “In Europe, native oyster restoration projects have been increasing over the past XYZ”. By providing the approximate time from which oyster restorations became more common, the authors will get at their earlier point in their original draft that it is a relatively new field that lacks data. | Revision accepted, rephrased to: “In the last decade, restoration of the European native oyster (Ostrea edulis) has become a common component of coastal conservation projects in Europe[1][8].” | 51 |
| 50 | Composition of what? Species composition? How is this different from biodiversity in many ways they are components of one another. Perhaps add in a reference that highlights the lack of knowledge on other reef functions (i.e. water filtration, habitat provisioning) and replace “composition” with whatever function you’d like to highlight along with biodiversity. | Revision accepted, rephrased to: “Due to the rarity of intact *O. edulis* reef habitats (e.g., [8][9][10][11]) there is a lack of data with regards to the biodiversity and structure of the faunal community associated with the reefs [2].” | 53 |
| 69-70 | Replace “to account for” with “mitigate the impacts of organic waste from the distillery’s discharge water”. | Revision accepted, rephrased to: “This initiative aims to restore native oysters to the Dornoch Firth whilst enhancing biodiversity and water quality in the system: the oyster filter feeding is expected to mitigate organic waste from the distillery’s discharge water.” | 77 |
| 90 | Revise. “importance” should be “important” | Revision accepted. | 100 |
| 109 | Revise. The revised sentence is still disjointed and hard to follow. What is an ‘ecological understanding’? The authors use this phrase frequently throughout but are mostly concerned with biodiversity. The authors could clarify by better communicating the link between biodiversity and ecosystem functioning. The language is very broad here and should be revised. | Revision accepted, rephrased to: “Oyster restoration is becoming a priority in many coastal conservation initiatives [14][8][35][36]. Many locations around Europe which supported historical oyster fisheries and /or remnant populations are now being considered as possible locations for *O. edulis* restoration [1][37][38]. Owing to the catastrophic decline and modern-day rarity of *O. edulis* reef habitats, and the historical context within which these declines occurred, it is extremely difficult to re-construct the biodiversity characteristics of a fully restored reef [39]. By examining the biodiversity of oyster habitats recovering from fishing activity[40], a greater understanding of the faunal community composition associated with oyster reef habitats can be gained [2][10][31] and used to manage expectations of biodiversity outcomes and progress towards biodiversity goals.” | 113 |
| 139 | Authors should state in the methods that the sampling was opportunistic. | Revision accepted, rephrased to: “Three treatments were selected opportunistically for survey in 2019…” | 142 |
| 143 | A supplemental figure of the transect survey layout would be helpful. | Revision accepted see S1 Figure. In-text citation added to line 148. | 148 |
| 217 | “Furthermore, the scale and trend in the observed relationships are consistent with successional differences (ADD CITATION HERE) not spatial patterns.” | Revision accepted. Added the following references: [10] Smyth & Roberts 2010 [25]Kaiser et al., 2006 [46] Scriberras et al., 2018 [47] Farinas-Franco & Roberts 2014 | 234 |
| Fig 3 | Add axis 1 and axis 2 labels as suggested by authors. | Revision accepted | Fig 3 |
| 217 | List/acknowledge potential confounding factors that were not co-monitored in this sentence “Potential confounding environmental factors (LIST HERE), were not included in the study.”. | Revision accepted, rephrased to: “Potential confounding environmental factors were not included in the study (eg depth, temperature and tidal flow rate) because they were judged to be diminutive.” | 230 |
| 217 | Explain what the authors mean by “out of proportion” with any environmental gradients? Also, this added language makes many sweeping statements without acknowledging what environmental gradients, factors, etc. may be at play. The authors should add specificity to the main text about the potential confounding factors that they did not evaluate. | Revision accepted, rephrased to: “Fishing is the dominant structural influence on the community, hence the results from the study are unlikely to be the result of environmental gradients that could produce such a significant scale of change. Furthermore, the scale and trend in the observed relationships are consistent with successional differences [10][25][46][47] not spatial patterns” | 234 |
| 219 | Add citations after “In keeping with most biodiversity studies,…” | Revision accepted. Added the following references: [44]Kent et al., 2017 [45] Benjamin et al., 2022 | 228 |
| 239 | Remove the word “consequential”. Revise “is potentially decadal in nature”--this language could be much improved. The authors should revise this section slightly for conciseness. The data supports that reef recovery begins within a year post disturbance and reaches reference levels within ten years. | Revision accepted, rephrased to: “Reef recovery likely begins as early as one year after fishing disturbance and increases over several more years as oyster spat settles and the size, density and complexity of oyster shell increases (Figure 8; Figure 9) [3][26][47]. Beyond the six-year timeline studied here the biodiversity trajectory is expected to plateau out, but examination of oysters in an undisturbed state would be required to confirm this.” | 254 |

In addition to the above comments, to improve readability and accuracy of statement further minor changes have been made:

Line 74: replacement of the word “*is*” with “*are*”

Line 93: Insert of term “*shellfish reefs*”

Line 94: deletion of word “*late*”

Line 107: replacement of the word “*is*” with “*are*”

Line 296: replacement of the word “*Therefore*” with “*Overall*”

Line 298: deletion of word “*Overall*”

In the acknowledgments line 134 has been added to reflect our gratitude for the referees feedback: “*The comments from the referees greatly improved the manuscript”.*

Furthermore, we have revisited text in lines 256-263 concerning intermediate disturbance and trophic cascades. We feel that the new edits more clearly address the reviewers’ previous comments and cover the topic more accurately. The section of text has been rephrased to: “*Elsewhere in longer term (10yr+) marine reserves studies, ‘trophic cascades’ have occurred when re-established higher trophic level species have restructured benthic communities beyond simple biodiversity and biomass increases that were initially observed [eg. 55]. A six year timeline in the present study is too short to exclude the possibility that trophic cascades might subsequently occur in oyster reef recovery. Attributing enhanced biodiversity to ‘intermediate disturbance’ [56] in the present study, however, seems unlikely because increasing niches as the reef undergoes physical structural recovery are unlikely to abate.*”

The following references were added to support the suggested revisions from the reviewer: Farinas-Franco & Roberts 2014 to line 217; Kent et al., 2017 and Benjamin et al., 2022 to line 219.

Likewise, references have been omitted from the manuscript because, due to revisions, they were no longer relevant: Haddad et al., 2015 from line 99; Graham & Nash 2013 from line 106 and 274.

Due to the changes to the references the in-text citations have been updated.

Finally a list of the supporting information captions has been added to the end of the manuscript in addition to relevant in-text citations and all figures have been processed through PACE and now meet PLOS requirements.

We look forward to hearing from you in due time regarding our submission and to respond to

any further questions and comments you may have.

Sincerely,

Naomi Kennon

10/12/22
