# Supplementary material for: Rotational fishing enables biodiversity recovery and provides a model for oyster (Ostrea edulis) habitat restoration
Source: PLoS One. 2023 Mar 29;18(3):e0283345. doi: 10.1371/journal.pone.0283345 (PMC10058151; doi:10.1371/journal.pone.0283345)
Supplement: Attachment — Submitted filename: Response to reviewers.docx [file pone.0283345.s007.docx]

Dear José A. Fernández Robledo, Ph.D. 24/01/23

Thank you for giving us an opportunity to submit a revised draft of our manuscript *Rotational fishing enables biodiversity recovery and provides a model for oyster (Ostrea edulis) habitat* *restoration* to PLOS One.

We again appreciate the time and effort that you and the reviewers have dedicated to providing valuable feedback on our manuscript. We are grateful to the reviewers for their insightful comments on the paper and have been able to incorporate these further changes to reflect the suggestions provided.

See the following table for a point-by-point response to the reviewers’ comments and concerns.

| **Line** | **Comment** | **Response** | **New line** |
| --- | --- | --- | --- |
| Abstract: In the last sentence | “indicate the likely scale of biodiversity gain” is unclear. The authors could consider replacing with “indicate the probable biodiversity benefits of oyster habitat restoration…”. The authors should also replace “tool” with “metric”, as shell density is a metric not a tool. | Revision accepted. Rephrased to “The findings from the present study indicate the probable biodiversity benefits of oyster habitat restoration and a cost-effective metric (shell density) to judge progress in restoration projects” | 36 |
| 40 | Rephrase; “Habitat restoration to enhance biodiversity is becoming…” | Revision accepted. Rephrased to “Habitat restoration to enhance biodiversity is becoming an increasingly popular conservation practice” | 41 |
| 42 | Delete “making them rare or endangered” or add a citation. | Revision accepted; line deleted. | 43 |
| 72 | Replace “bottom-contacting fishing gear” with “Bottom towed fishing gear” to match the following sentence. | Revision accepted. Rephrased to “towed” | 74 |
| 74 | If correct, the better reflect the destruction of these practices, could consider replacing “some” with “many”. | Revision accepted. Rephrased to “damages and kills many benthic organisms” | 76 |
| 77 | Replace “with” with “and a significant reduction in biodiversity” | Revision accepted. Rephrased to “and a significant reduction in biodiversity” | 80 |
| 79-81 | Need citation | Added [2][16][17][26]. | 83 |
| 85 | Replace “around” with “in” | Revision accepted. | 87 |
| 92 | Replace “disruption” with “disturbance” | Revision accepted. | 94 |
| 97-98 | Remove “From 2021” and start the sentence with “The UN Decade on…” | Revision accepted. | 100 |
| 101 | Replace restoration projects with restoration practitioners since you’re naming entities/stakeholders in this sentence. | Revision accepted. | 104 |
| 119 | Hypothesis 1 does not address the live vs. dead shell component described above. This should be integrated into the hypothesis. | Revision accepted. Rephrased to “The macrofaunal biodiversity of an oyster reef community is affected by the density of live and dead oyster shells.” | 124 |
| 135 | I recommend removing “geographic gradient” as this would suggest there are environmental conditions that vary along that gradient (different reef rigosity, depth, etc. could impact flow, temperature, light penetration). If the authors are convinced that environmental conditions were similar across transects, it’d be best to remove “geographic gradient” and have the sentence read as “Sampling was conducting along X (put number of transects here) 25 m transects that ran parallel to shore.” | Revision accepted. Rephrased to “Sampling was conducted along a total of twelve 25m transects that ran parallel to the shore.”  Following this change in the next line, the word “A” was replaced with “Each” to improve readability. | 139 |
| 143-145 | Did the divers count the shells in the field or was all counting down using ImageJ--please clarify. | Revision accepted. Rephrased to “In each photo quadrat, the number of oyster shells was counted using the software ImageJ v1.52 by tracing around the shape of the individual shells” | 147 |
| 154 | Replace “within” with “by” | Revision accepted. | 158 |
| 164 | Should MDS be nMDS? | Although “MDS” is often used in a general sense to include non-metric MDS, we accept that “nMDS” is technically more precise for our analyses. Revision accepted (also at line 188). | 171 |
| 172-173 | Rephrase to - (“diminishing rate of increase as reef approaches ‘recovered’ condition”) | Revision accepted. Rephrased to “(diminishing rate of increase as reef approaches ‘recovered’ condition).” | 179 |
| 173 | The authors should explain why year was log-transformed. | Log-transformation of year was used to reflect the non-linear nature of the recovery process, representing it as a logarithmic function of time. Sentence revised to “The response variable was modelled as a function of the log of years since disturbance to represent this non-linear (logarithmic) process.” | 181 |
| 177-178 | Should be in methods | Revision accepted line moved to 152. “Video footage was taken of the seafloor covering a 0.5m swath on each side of the transect, representing a total of approximately six hours of footage.”  Remaining line rephrased to “A total of 27 macrofaunal species were recorded in the course of the surveys (S2 Table & S3 Table).” | 152 |
| 181 | Replace “a very good” with “is an accurate representation” | Revision accepted | 189 |
| 192 | Table 1 -- Referring now to the treatments and treatment sites is confusing to the reader, I suggest maintaining the naming system above and referring to Treatments 1 and 3 (removing “site”). | Revision accepted | 200 |
| 195-197 | It’d be great if in the methods, the authors wrote a quick few sentences about the strengths and weaknesses of Shannon-Wieners, Margalef’s and Pielou’s evenness so the reader can better make sense of the results and what each of these values means. | Revision accepted. Rephrased to “including calculation of the biodiversity indices: Margalef’s richness (d) a measure based on the number of species present and sampling effort; Pielou’s evenness (J) a measure of how evenly species are distributed; and Shannon-Wiener’s diversity (H’) which takes both richness and evenness into account.” | 165-167 |
| 198 | Average oyster shell density increased between what treatments? Only 1 and 3? Need to specify. | Revision accepted. Rephrased “increased between treatments one and two by 749.5%” | 204 |
| 217 | Rephrase--“…is expected to represent community-wide patterns” | Revision accepted. Rephrased “the sampling method presented here is expected to represent community-wide patterns.” | 223 |
| 218 | This is great, I think it’s really important to acknowledge the data that wasn’t collected for this study and could have (but are unlikely to have) influenced results. Instead of “included in the study” replace with “were not evaluated”. | Revision accepted. Rephrased “Potential confounding environmental factors were not evaluated” | 224 |
| 230;233 | Replace bottom-contacting with bottom towed. Unless the authors are referring to other fishing practices (shrimp pots, lobster traps, bottom trawling)--if this is the case, some examples in parentheses would be helpful. | Revision accepted | 237;240 |
| 246 | Rephrase to “…that trophic cascades could ultimately transform the benthic community and affect oyster reef recovery” | Revision accepted. Rephrased to “A six year timeline in the present study is too short to exclude the possibility that trophic cascades ultimately transform the benthic community and affect oyster reef biodiversity.” | 256 |
| 247-249 | I suggest the authors improve the text in this sentence here as it’s unclear what they are trying to say is the reasoning behind why their study is not evidence of intermediate disturbance hypothesis. Also, intermediate disturbance hypothesis appears out of nowhere so perhaps the authors could add a sentence before the current one that addresses other potential explanations to support their findings and then follow it up with why it doesn’t apply (as they’ve done, but with slight editing to make clearer). | On reflection we believe this point is far too speculative and have remove the sentence. The paragraph therefore concludes on the far more persuasive consideration of trophic cascade. | 256-257 |
| 250 | What kind of relationship? Positive? Negative? This type of information is important for guiding the reader. | Revision accepted. Rephrased to “Logarithmic regression showed positively correlated relationships between biodiversity, oyster shell density and time since disturbance.” | 258 |
| 250-258 | The authors should indicate the directionality of all of the relationships in this paragraph to help guide the reader. | Revision accepted. Rephrased to “Logarithmic regression showed positively correlated relationships between biodiversity, oyster shell density and time since disturbance. Oyster shell density was the best statistical predictor of Shannon-Wiener’s diversity, which is likely to be due to the positive relationship between shell density and structural complexity [11][13]. Lown et al. (2021) demonstrated a positive relationship between oyster density and biodiversity using dredge sampling in a remnant oyster fishery in England [11]. The present findings are consistent with Lown et al. (2021), confirming a strong relationship between biodiversity and shell density [11].” | 258-264 |
| 260-263 | Great! |  |  |
| 278 | Rephrase slightly: “perhaps the most direct evidence yet quantifying the potential biodiversity gain…” | Revision accepted. Rephrased to “The relationship between oyster shell and biodiversity presented here is perhaps the most direct evidence yet quantifying the potential biodiversity gain” | 286 |
| 280 | Replace “would probably double in a decade” to “would likely double within a decade” | Revision accepted. Rephrased to | 289 |
| 281 | Make more concise; “the present findings demonstrate the benefits of restoration and the trajectory of restoration success as it relates to biodiversity.” Then, the authors could start a new sentence “Our data showed a link between increased shell material and increased biodiversity. | Revision accepted. Rephrased to “Overall, the present findings demonstrate the benefits of restoration and the trajectory of restoration success in terms of biodiversity [1][35][36]. Our data showed a link between increased shell material and increased biodiversity.” | 290 |
| 283 | Missing a period. | Revision accepted. | 293 |
| 284 | Rephrase; “rare example from a long-term dredge fishery whose practices appear to have allowed rare oyster habitat and the associated community to persist, thereby providing a valuable insight into the recovery of biodiversity by balancing sustainable fishery practices with European oyster habitat recovery efforts”. Since this study did not track restoration over time but recovery post disturbance, distinguishing between the two is critical. | Revision accepted. Rephrased to “The present study is a rare example from a long-term dredge fishery [40][48] whose practices appear to have allowed rare oyster habitat and the associated community to persist, thereby providing a valuable insight into the recovery of biodiversity by balancing sustainable fishery practices with European oyster habitat recovery efforts.” | 294 |

The following change was also made:

Figure caption 1. “Lefnol point” was corrected to “Lefnol Point”

We look forward to hearing from you in due time regarding our submission and to respond to

any further questions and comments you may have.

Sincerely,

Naomi Kennon

24/01/23
